# Occurrence of *Encephalitozoon cuniculi* and *Encephalitozoon hellem* in European Wild Rabbits (*Oryctolagus cuniculus*) in Southern Germany (Bavaria)

**DOI:** 10.3390/ani14192880

**Published:** 2024-10-07

**Authors:** Katharina Breuninger, Monika Rinder, Rüdiger Korbel

**Affiliations:** Center for Clinical Veterinary Medicine, Clinic for Birds, Small Mammals, Reptiles and Ornamental Fish, Veterinary Faculty, University of Munich, 85764 Oberschleissheim, Germany; monika.rinder@lmu.de (M.R.); korbel@lmu.de (R.K.)

**Keywords:** microsporidia, wildlife, Lagomorpha, zoonotic, IFAT, PCR

## Abstract

**Simple Summary:**

Microsporidia are a group of fungus-related eukaryotes with worldwide distribution. The microsporidian species *Encephalitozoon cuniculi* and *E. hellem* occur in mammals, birds and even humans. Knowledge of their relevance in wild rabbits is very limited so far. Thus, the aim of the present study was to investigate the occurrence of *E. cuniculi* and *E. hellem* in wild rabbit populations in southern Germany (Bavaria). Therefore, blood and organ samples of 158 wild rabbits were investigated by serological and PCR-based assays. Antibodies to *E. cuniculi* were detected in 24 of the 158 (15.2%) wild rabbits, while DNA of *E. cuniculi* was found in 7 (4.4%) and DNA of *E. hellem* was found in 3 (1.9%). Sequencing identified *E. cuniculi* genotype 1. This study provides the first *E. cuniculi* genotype determination in free-living wild rabbits worldwide and the first evidence of *E. hellem* in rabbits worldwide. Wild rabbits should, therefore, be regarded as a reservoir for both pathogens and as a source of infection for domestic rabbits, other animals and humans.

**Abstract:**

*Encephalitozoon cuniculi* and *Encephalitozoon hellem* are fungus-related, obligate intracellular pathogens belonging to the microsporidia. Both microorganisms occur in mammals, birds and even humans, thus revealing a zoonotic potential. Knowledge of their relevance in wild rabbits is very limited so far. Thus, the aim of the present study was to investigate the occurrence of *E. cuniculi* and *E. hellem* in wild rabbit populations in southern Germany (Bavaria). Therefore, blood and organ samples (brain/kidney) of 158 wild rabbits were investigated by immunofluorescence and PCR-based assays. Antibodies to *E. cuniculi* were detected in 24 of the 158 (15.2%) wild rabbits, while DNA of *E. cuniculi* was found in 7 (4.4%) and DNA of *E. hellem* was found in 3 (1.9%). Sequencing identified *E. cuniculi* genotype 1. This study provides the first *E. cuniculi* genotype determination in free-living wild rabbits worldwide and the first evidence of *E. hellem* in rabbits worldwide. Wild rabbits should, therefore, be regarded as a reservoir for both pathogens and, on the basis of molecular evidence from kidney tissue and presumed urine excretion, also as a source of infection for *E. cuniculi* for animals and humans.

## 1. Introduction

*Encephalitozoon cuniculi* and *Encephalitozoon hellem* are obligate intracellular, spore-forming pathogens belonging to the group of microsporidia. *E. cuniculi* was first described by Wright and Craighead in 1922. They discovered it by chance during experimental research as the causative agent of motor paralysis in young rabbits [1]. The pathogen is mainly associated with rabbits, but it has a very broad host range and may occur in many other mammals including humans, as well as in birds [2,3,4].

So far, four different genotypes of *E. cuniculi* have been identified, which may be differentiated based on molecular genetic investigations of the ITS region of the rRNA gene. Those genotypes are called “rabbit strain” (type I), “mouse strain” (type II), “dog strain” (type III) and “human strain” (type IV) [5,6], but are of low host-specificity. For example, in rabbits, natural infections with genotypes I, II and III have been detected [7,8]. In humans, in addition to further genotype IV [6], infections with all of the other three genotypes were also identified, revealing a zoonotic potential independent of the genotype involved [3,9].

Horizontal infections with *E. cuniculi* occur primarily through oral ingestion of spores, which are excreted intermittently by infected animals or humans, mainly via the urine but also with faeces [10,11]. The oral route with ingestion of contaminated food and water is the most probable route of transmission of microsporidian spores to humans [3,12].

Infections with *E. cuniculi* in rabbits are usually clinically asymptomatic, but immunosuppression may lead to clinical signs and even death [13]. After ingestion, the spores enter the intestine, where they infect the epithelium and then reach the bloodstream via the gut-associated lymphoid tissue. Via the blood, the pathogen reaches various organs—either as a free spore or within infected monocytes [14]. The final predilection sites are the brain and the kidneys [10]. In addition, ocular transmission of the pathogen during the intrauterine period is possible [15]. Cerebral infection typically results in non-suppurative, focal to multifocal granulomatous (meningo-) encephalitis [16,17,18,19]. In the kidneys, *E. cuniculi* multiplies primarily in the tubular epithelial cells. Histologically, the kidneys typically show focal, multifocal or segmental non-suppurative granulomatous interstitial nephritis with macroscopic scarring due to fibrosis [17,18,19,20,21]. Clinically, infections manifest in neurological symptoms (vestibular dysfunction, e.g., head tilt, ataxia, nystagmus, rotations around the longitudinal axis), signs of renal insufficiency (non-specific symptoms such as apathy, anorexia, weight loss, polydypsia, polyuria) or ocular signs (phacoclastic uveitis, consecutive cataracts) [22,23,24]. Clinical manifestation in humans usually occurs in immunocompromised individuals only. Diarrhea and abdominal pain, hepatitis, peritonitis, liver failure, renal failure, disseminated disease with fever, persistent cough and endocarditis have been described [12].

*E. hellem* was first described in humans in 1990 as a cause of keratoconjunctivitis in AIDS patients [25,26]. Morphologically and ultrastructurally, *E. hellem* and *E. cuniculi* do not differ. However, immunological and molecular testing methods make it possible to distinguish between the two species [25]. In animals, *E. hellem* is most widespread among birds, both wild and companion birds, but the pathogen has also been occasionally detected in mammals, including rodents, carnivores and monkeys [4]. In Lagomorpha, natural infection with *E. hellem* has only been detected in the kidney tissue of a single European brown hare so far, which was simultaneously infected with *E. intestinalis* [27].

Three different genotypes (genotypes 1, 2 and 3) of *E. hellem* have been described by Mathis et al. based on molecular genetic analyses of the ITS region of the rDNA gene [28]. Subsequently, Xiao et al. were able to subdivide genotype 1 into 1A, 1B and 1C and genotype 2 into 2A and 2B by additionally analysing the gene locus of the polar tube protein and the gene of the small subunit of the rRNA; it has been recommended to rename genotype 3 as 2C [29].

*E. hellem* infections in birds may cause a variety of clinical symptoms ranging from mild to fatal [30,31]. However, most infections are probably asymptomatic [32,33]. Increased stunting and high mortality have been observed in budgerigar chicks [30]. Necropsy of infected birds revealed abnormalities such as significant muscle wasting, loss of body fat and lesions, especially in the kidneys, liver, intestines and eyes [34]. Clinical manifestations in humans, as with *E. cuniculi*, occur predominantly in immunocompromised individuals. Keratoconjunctivitis in particular, but also nephritis, pneumonia, bronchitis and disseminated disease with renal failure have been reported [12].

Since hunted European wild rabbits (*Oryctolagus cuniculus*) in Germany and elsewhere are partially intended for consumption and usually enter the human food chain without prior hygiene control in accordance with country-specific legislation, there is a need to identify possible zoonotic agents such as *E. cuniculi* and *E. hellem*. In the past, the serological prevalence of *E. cuniculi* in domestic rabbits was found to vary between 7.7 [35] and 81.7% [36] worldwide. However, significantly fewer studies have been conducted in wild rabbits, and there is no current data on *E. cuniculi* and a complete lack of data on *E. hellem* for Central Europe. Previous serologic studies on *E. cuniculi* in Germany on wild rabbits have shown contradictory results. The first study in southern Germany in 1988 showed a seroprevalence of 18.1% in 155 wild rabbits investigated [37], but in a subsequent study in northern Germany in 1996, none of 100 wild rabbits tested positive [38]. Molecular studies to detect the nucleic acids of the pathogens had not yet been carried out in wild rabbits in Germany so far.

The aim of this study was, therefore, to gain updated insight into the prevalence of *E. cuniculi* using serological and molecular assays and to investigate the occurrence of *E. hellem* in wild rabbits in Germany for the first time in order to assess whether wild rabbits represent a reservoir for both pathogens and thus a potential source of infection.

## 2. Materials and Methods

### 2.1. Ethics

The Ethics Committee of the Faculty of Veterinary Medicine at LMU Munich approved this study, and no ethical concerns were raised (reference number 317-30-06-2022). Most of the samples were collected from legally hunted wild rabbits during the hunting seasons. Three samples originated from wild rabbits from a shelter that died or had to be euthanised due to severe disease independently of the current investigation. One of the samples came from a wild rabbit that had been found freshly dead, presumably after an accident with a car.

### 2.2. Study Area and Sample Collection

**Figure 1:**
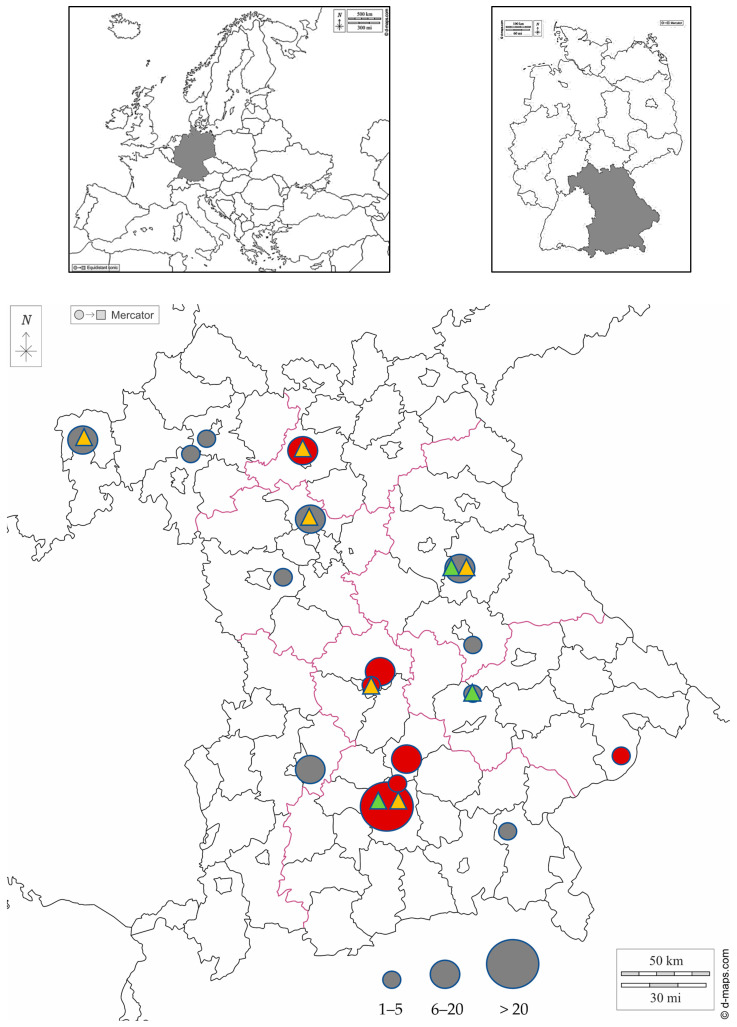
Rural administrative district map of Bavaria showing the collection areas of wild rabbits. Numbers of sampled rabbits per administrative district are semiquantitatively indicated by differing circle diameters. Areas in which wild rabbits were tested seropositive for *E. cuniculi* are marked in red, while areas in which all tested wild rabbits were seronegative are marked in dark gray. Areas in which *E. cuniculi* DNA has been detected in wild rabbits using molecular testing methods are highlighted with a yellow triangle. Areas in which *E. hellem* DNA has been detected using molecular testing methods are highlighted with a light green triangle. Maps were taken from d-maps.com (https://d-maps.com/carte.php?num_car=6121&lang=de, https://d-maps.com/carte.php?num_car=17879&lang=de, https://d-maps.com/carte.php?num_car=2233&lang=de; accessed on 1 August 2024).

This study was conducted between 2021 and 2023. A total of 158 European wild rabbits (*Oryctolagus cuniculus*) originating from all seven administrative districts of Bavaria, a federal state located in the south of Germany, were sampled (Figure 1). Data on age (juvenile (≤200 days [39], not fully grown)/adult), gender, location of origin and sampling year were recorded for each wild rabbit included in the investigation (Table 1, Supplementary Table S1).

Brain, kidney and blood samples were taken from each individual. Only rabbits with both blood and tissue samples available were included in this study. Sampling was either performed by the hunters themselves immediately after the hunt according to previous instructions, or the carcasses were provided whole and tissue and blood samples were taken in the laboratory. The fresh clinical samples or the carcasses were generally submitted to the laboratory within 1–3 days. For 46 of the 158 carcasses/samples, however, no immediate transfer could take place due to logistical circumstances, thus they were frozen at −18 °C by the hunters in the meantime.

Tissue samples were placed in sterile plastic containers (125 mL) and blood samples were collected in 4 mL serum tubes. The tissue samples were stored at −20 °C until analysis. The serum tubes were centrifuged after clotting, and the supernatant was pipetted off and transferred to sterile 1.5 mL Eppendorf tubes. The Eppendorf tubes containing the serum samples were stored at −20 °C until used for *E. cuniculi* antibody testing.

### 2.3. Indirect Immunofluorescence Antibody Test

The indirect immunofluorescence antibody test (IFAT) for the detection of *E. cuniculi* antibodies was carried out according to Chalupsky et al. [40] by a commercial company (SYNLAB.vet GmbH, Augsburg, Germany). Both IgG and IgM antibodies were tested. The highest serum dilution level evaluated as positive was given as a titer. Titers of ≥1:80 were considered positive for both antibody isotypes. Possible associations between seropositivity and the age or gender of the rabbits were verified using the Chi-Square test.

### 2.4. DNA Extraction

A total of 500 mg of brain or kidney tissue was added to 150 µL of sterile phosphate-buffered saline (pH 7.2) and then homogenised with the aid of SiLibeads Typ ZS 1.4–1.6 mm (Sigmund Lindner GmbH, Warmensteinach, Germany) using a vibrating mill (Retsch, Haan, Germany) for 5 min with a frequency of 30 Hz. A total of 200 mg of the homogenised tissue material was weighed into a microcentrifuge tube, mixed with 600 µL of sterile phosphate-buffered saline (pH 7.2) by pulse-vortexing and then clarified by centrifugation at 16,200× *g* for 2 min. Nucleic acid was extracted from 200 µL of the supernatant using the IndiSpin^®^ Pathogen Kit (Indical Bioscience GmbH, Leipzig, Germany) according to the manufacturer’s instructions, except that proteinase K digestion was performed at 56 °C for one hour instead of 20–25 °C for 15 min.

### 2.5. Real-Time PCR

The extracted DNA was tested for *E. cuniculi* and *E. hellem* in a duplex real-time PCR analysis, as described before by Leipig et al. [41], using the *E. cuniculi* forward primer MSP-3 (5′-TTGCGATGAAGGACGAAGG-3′), the *E. hellem* forward primer MSP-4 (5′-TGATGAAGGACGAAGG-3′), a reverse primer (MSP-5: 5′-TCTTGCGAGCGTACTATCC-3′) and the molecular beacon fluorescent probes for *E. cuniculi* (MSP-S3: 5′-FAM-CGCGATCGACTGGACGGGACNGTGTGTGTTGTCCATGAGAAAGATCGCG-BHQ-1-3′) and *E. hellem* (MSP-S4: 5′-HEX-CGCGATCGACTGGACGGGACTGTTTTAGTGTTGTCCGAGAGAAAGATCGCG-BHQ-1-3′). Primers and probes were synthesised by Metabion (Planegg, Germany).

Real-time PCR was performed using 2.5 u AllTaq DNA polymerase (Qiagen, Hilden, Germany), 0.5 μM MSP-3, 0.5 µM MSP-4, 1 μM MSP-5, 0.5 μM MSP-S3, 1 µM MSP-S4, 0.25 mM, each, dNTPs, 2.5 μg bovine serum albumin, 5.5 mM MgCl_2_, 1× key buffer (Qiagen, Hilden, Germany) and 2.5 μL of DNA in a total volume of 25 μL. The real-time PCR assay was performed using a G8830A AriaMx Real-time PCR System (Agilent Technologies, Santa Clara, CA, USA) using an initial denaturation for 15 min at 95 °C, followed by fifty cycles of denaturation for 30 s at 95 °C, annealing for 60 s at 53 °C and elongation for 30 s at 72 °C. Samples that yielded Ct values calculated by the Aria data analysis software and revealed a sigmoid shape of the fluorescence curve were then further analysed in conventional nested PCR assays for confirmation and genotyping.

### 2.6. Nested PCR I

In order to confirm the results of the real-time PCR assay obtained for *E. cuniculi* and *E. hellem* and thus to identify the species, a nested PCR I was performed according to Katzwinkel-Wladarsch et al. [42] with minor modifications. The primers used were generic microsporidia primers. For the first round, forward primer MSP-1 (5′-TGAATGKGTCCCTGT-3′) and reverse primer MSP-2a (5′-TCACTCGCCGCTACT-3′) were applied, and in the second round, forward primer MSP-3 (5′GGAATTCACACACCGCCCGTCRYTAT-3′) and reverse primer MSP-4a (5′-CCAAGCTTATGCTTAAGTYMAARGGGT-3′). The primers were synthesised by Metabion (Planegg, Germany).

The first PCR round contained 1.25 u AllTaq DNA polymerase (Qiagen, Hilden, Germany), 0.5 μM of each respective primer, 0.25 mM, each, dNTPs, 1.0 mM MgCl_2_, 1× key buffer (Qiagen, Hilden, Germany) and 2.5 μL DNA in a total volume of 25 μL.

The second PCR round contained 1.25 u AllTaq DNA polymerase (Qiagen, Hilden, Germany), 0.5 μM of each respective primer, 0.25 mM, each, dNTPs, 0.5 mM MgCl_2_, 1× key buffer (Qiagen, Hilden, Germany) and 1 μL of the first PCR round in a total volume of 25 μL. The nested PCR assay was performed using a SensoQuest LabCycler (SensoQuest GmbH, Göttingen, Germany). For both rounds, identical temperature profiles were used. The initial denaturation was carried out for 2 min at 96 °C, followed by fifty cycles of denaturation for 60 s at 92 °C, annealing for 60 s at 58 °C, elongation for 90 s at 72 °C and a final extension step for 7 min at 72 °C.

PCR products were visualised under UV light after electrophoresis in 2% agarose gels with ethidium bromide.

### 2.7. Nested PCR II

The nested PCR II aimed at determining the genotypes of *E. cuniculi* involved. All samples for which real-time PCR had previously yielded Ct values for *E. cuniculi* were included. The first round of the nested PCR assay was performed according to Asakura et al. [43] with minor changes, using the *E. cuniculi* forward primer F2 (5′-TCCTAGTAATAGCGGCTGAC-3′) and the *E. cuniculi* reversed primer int580r (5′-TTTCACTCGCCGCTACTCAG-3′), which was originally designed by Didier et al. [5]. Primers were synthesised by Metabion (Planegg, Germany). The PCR reaction contained 0.625 u AllTaq DNA polymerase (Qiagen, Hilden, Germany), 0.2 μM of each primer, 0.2 mM, each, dNTPs, 1× key buffer (Qiagen, Hilden, Germany) and 2.5 μL of DNA in a total volume of 25 μL. The PCR assay was performed with an initial denaturation for 2 min at 95 °C, followed by thirty-five cycles of denaturation for 30 s at 94 °C, annealing for 30 s at 56 °C, elongation for 30 s at 72 °C and a final extension step for 5 min at 72 °C.

For the second round of nested PCR II *E. cuniculi,* forward primer EcF3 (5′-AAGATGACGCACTGGACGAA-3′) and reverse primer EcR3 (5′-GTGCACACCGCACACAATTC-3′) were designed for the present study based on the identification of rDNA genomic regions conserved for the varying *E. cuniculi* genotypes. Primers were synthesised by Metabion (Planegg, Germany). The PCR reactions contained 0.625 u AllTaq DNA polymerase (Qiagen, Hilden, Germany), 0.25 μM of each primer, 0.2 mM, each, dNTPs, 1× key buffer (Qiagen, Hilden, Germany), 1 μL of the first PCR round in a total volume of 25 μL. The initial denaturation was carried out for 2 min at 95 °C, followed by forty cycles of denaturation for 5 s at 95 °C, annealing for 15 s at 53 °C and elongation for 10 s at 72 °C.

PCR products were visualised under UV light after electrophoresis in 2% agarose gels with ethidium bromide.

### 2.8. Sequencing

PCR products of expected sizes were extracted from agarose gel using the QiaQuick gel extraction kit (Qiagen, Hilden, Germany) and sequenced by Sanger’s method at Eurofins Genomics (Ebersberg, Germany) from both sides, using the respective PCR primers of the second PCR rounds. The BLAST search tool provided by the National Centre for Biotechnology Information (http://blast.ncbi.nlm.nih.gov/Blast.cgi; access date: 3 May 2024) was used to determine the identity of the PCR products. To determine the genotype of *E. cuniculi* sequence alignments were carried out using CLUSTAL W, which is included in the DNAMAN software package (Lynnon Corporation, Quebec, QC, Canada), including sequences for the different *E. cuniculi* genotypes deposited in the NCBI GenBank.

## 3. Results

### 3.1. Indirect Immunofluorescence Antibody Test for E. cuniculi

Serum samples obtained from 24 of the 158 wild rabbits (15.2%) were positive for antibodies against *E. cuniculi*. No association was found between seropositivity and age (X^2^ (1, N = 158) = 1.116, *p* = 0.29)) or gender (X^2^ (1, N = 158) = 0.06, *p* = 0.80)) using the Chi-Square test.

In 12 of the samples, both IgM and IgG antibodies were found. As shown in Table 2, only IgG antibodies were detected in 10 samples, while only IgM antibodies were found in 2 samples. In 19 serum samples, low titres of 1:80 were detected. Higher titres ranging from 1:160 to >1:1280 were found in five samples (Table 2). Wild rabbits originating from 7 of the 17 locations in Bavaria included in this study tested positive (Table 2, Figure 1).

### 3.2. Genome detection by Real-Time PCR

Real-time PCR revealed Ct values for *E. cuniculi* in 10 of the 158 (6.3%) and for *E. hellem* in 4 of the 158 (2.5%) wild rabbits (Supplementary Tables S2 and S3). The Ct values for *E. cuniculi* ranged between 27.67 and 45.98, and those for *E. hellem* were between 42.93 and 48.23. Only the Ct values of those samples for which the curves showed a sigmoid shape were taken into account.

Positive reactivities for *E. cuniculi* were found in both the brain and the kidneys of 2 rabbits (Nos. 9 and 118) as well as in the brains only of 3 rabbits (Nos. 75, 79 and 155) and in the kidneys only of 5 rabbits (Nos. 66, 68, 97, 134 and 157) (Supplementary Table S2).

Regarding the 4 rabbits with Ct values for *E. hellem*-positive reactions, 1 rabbit (No. 118) originated from both the brain and kidney samples, while in 2 rabbits (Nos. 97 and 150), the kidney samples only, and in 1 rabbit (No. 109) the brain sample revealed fluorescence signals (Supplementary Table S3).

A total of 2 of the rabbits showed Ct values for both *E. cuniculi* and *E. hellem*, 1 rabbit (No. 118) in the brain and kidney samples and the other (No. 97) in the kidney sample.

### 3.3. Nested PCRs and Sequencing

Samples from rabbits that gave Ct values in the real-time PCR were subsequently analysed with nested PCRs for confirmation and genotyping.

The nested PCRs I yielded PCR products of the expected size for *E. cuniculi* in 7 of the 10 rabbits that had previously yielded Ct values in the real-time PCR (Supplementary Table S2, Supplementary Figure S1). For *E. hellem*, products of the expected size were obtained in three of the four rabbits that previously gave Ct values in the real-time PCR (Supplementary Table S3, Supplementary Figure S2).

Using nested PCR II, in 1 rabbit (No. 9), *E. cuniculi* genotype 1 characterised by three 5′-GTTT-3′ repeats in the ITS DNA sequence, [5] was found. Identical sequences were obtained from both brain and kidney samples of this rabbit. The sequences are available in GenBank under the accession numbers PQ214196 and PQ214197. For the remaining PCR products of nested PCR I and II, including those of *E. hellem*, direct Sanger sequencing did not result in evaluable nucleotide sequences, probably because of the low DNA amount or bad DNA quality of the PCR product. The genotype of *E. hellem* involved could thus not be determined.

## 4. Discussion

In the current study, antibodies against *E. cuniculi* were detected in 15.2% of the wild rabbits tested, which is comparable to the results of the first investigation in southern Germany [37]. In that previous study, published in 1988, 18.1% of the wild rabbits tested positive. However, the wild rabbits analysed at that time came from only two properties in Munich, which limited the significance of the results [37]. The current results show that the pathogen is circulating in various areas of Bavaria and, considering the former study, indicate the endemic occurrence of *E. cuniculi* in the wild rabbit population, at least in southern Germany. Whether this endemic occurrence also extends to other regions in Germany is still unclear, as in another study including wild rabbits from the northern part of Germany published in 1996, antibodies against the pathogen were not detected, and it was therefore assumed that wild rabbits probably do not play a role in the spread of the pathogen [38]. Further new investigations are thus necessary to answer this question.

In the present study, *E. cuniculi* genomic DNA was detected in the examined animals in addition to the presence of antibodies. The current investigation thus represents the first successful molecular detection of *E. cuniculi* genomes from organ material of free-living wild rabbits worldwide and demonstrates that the wild rabbits examined were not only exposed to the pathogen but were actually infected. In 10 of the 158 (6.3%) wild rabbits, Ct values for *E. cuniculi* were obtained in the real-time PCR assay used. These results were subsequently confirmed by nested PCR in 7 out of the 10 rabbits. In addition, the detection in the kidney material, in particular, indicates that wild rabbits are not only a reservoir of the pathogen but, as has been proven in domestic rabbits already [44] are most likely also a source of infection due to the potential excretion of spores via the urine. Some rabbits within the present investigation showed Ct values by real-time PCR and did not reveal detectable antibody titres in the IFAT at the same time. Similar results of lacking antibody detections have been reported already in the past [45,46]. The potential reasons may only be speculated. Besides false-negative IFAT results caused by individual serum-inherent inhibition and false-positive PCR results, which were excluded as far as possible in the current study by a further confirmatory PCR test, causes may be an as-yet undetectable antibody level, an insufficient amount of spores ingested for seroconversion [45] or immunosuppressive effects of other diseases that prevent antibody production [45,46]. Excessive binding of antibodies by the pathogen could also play a role [46].

Up to the investigation presented here, there have been three prevalence studies on *E. cuniculi* in European wild rabbits based on DNA testing, all of which were conducted in Spain (including Tenerife). In addition, molecular methods have been used in a prevalence study of Eastern cottontail rabbits in Italy [47]. In one of the three studies from Spain, *E. cuniculi* DNA was detected by PCR in 2 of the 50 faecal samples examined [48]. However, the positive faecal samples originated from wild rabbits temporarily housed on farms in close contact with each other, which means that there might have been an increased risk of transmission. In the same study, *E. cuniculi* could not be detected in faecal samples originating from free-living wild rabbits, but other unknown microsporidian species were detected in five of these rabbits [48]. In the two other studies from Spain (0%, 0/383 [49], 0/438 [50]), DNA from *E. cuniculi* could not be detected [49,50]. However, only kidney tissue [49] or faecal samples were analysed [50]. In a former investigation conducted in Italy [47] on Eastern cottontail rabbits, a lagomorph species introduced for hunting purposes from North America to Italy in the 1960s, *E. cuniculi* was detected from organ tissues by conventional PCR in almost 10% of individuals that were tested. In this study, in addition to the brain and kidneys, the pathogen was also detected in skeletal muscle [47], which provides an interesting finding with regard to the potential risk of infection for humans and animals through the consumption of muscle meat. In histopathological studies on 34 European wild rabbits from Spain [51] and 62 European wild rabbits from England [52], no spores of *E. cuniculi* were detected in any of the tissue samples examined. In the study carried out in Spain, a broad range of organs of the wild rabbits were screened [51]. In the study carried out in England, only the kidneys were screened [52]. In both studies, the tissue samples were stained with hematoxylin and eosin (HE) and examined using light microscopy [51,52].

*E. cuniculi* genotype 1 (“rabbit strain”) was detected in both the brain and kidney tissue of one rabbit in the present study. This represents the first description of *E. cuniculi* genotypes in free-living wild rabbits. In previous studies on wild rabbits, genotyping of *E. cuniculi* was only carried out in one study conducted in Spain from faecal samples. Genotype 1 was also detected in this study, but only in wild rabbits temporarily housed on farms, which may have an increased risk of infection and other possible sources of infection compared to those in the wild [48].

Studies in wild rabbits in which both antibody determination and molecular methods were used for comparison were not available so far. Our study allowed for the first time a comparison of both antibody determination and PCR testing to assess whether wild rabbits are affected by the pathogen and thus pose a potential risk of infection to other animals and humans.

Antibodies against *E. cuniculi* were detected in 24 of the 158 wild rabbits in the current study, in 12 rabbits with both IgG and IgM, in 10 rabbits with exclusively IgG and in 2 rabbits with exclusively IgM (Table 2). Most prevalence studies, both in domesticated and wild rabbits, are based on the detection of antibodies, partly because this investigation can easily be performed antemortem. The interpretation of antibody detection is difficult, as it basically only proves exposure to the pathogen in the first place [53]. Simultaneous testing of IgG and IgM antibodies can provide an indication of infection status. The exclusive detection of antibodies of the IgM isotype serves as an indication of an early, acute infection. If both IgM and IgG isotype antibodies are detected, this indicates an acute infection. If only antibodies of the IgG isotype are present, this is an indication of a latent/chronic infection [10,54,55]. In the current study, solely IgM antibodies were detected in two of the wild rabbits examined, suggesting an early infection. Previous antibody determinations in wild rabbits were most often tested only for IgG antibodies, possibly leaving early-stage infections undetected.

Previous global serological studies on *E. cuniculi* in wild rabbits reported seroprevalences ranging from 3.9 to 100% in the UK (100%; 3/3) [56], Germany (18.1%, 28/155) [37], France (3.9%; 8/204) [57], Australia (24.7%; 20/81) [58] and Slovakia (44.7%; 21/47) [59], but no seropositive rabbits at all were found in further studies in Australia (0%, 0/823) [60], New Zealand (0%, 0/57) [60], the UK (0%, 0/175 [61]; 0/27 [62]; 0/60 [63]) and Germany (0%, 0/100) [38], suggesting that wild rabbits do not generally serve as a reservoir for the pathogen. In comparison to these varying results in wild rabbits, antibodies are regularly observed in domestic rabbits, especially pet rabbits, and often with high prevalence [64]. Due to the primarily high seroprevalences among domestic rabbits, some authors have suggested that the pathogen may be transmitted from domestic rabbits to wild rabbits [38,65]. In contrast, other researchers even suspect that wild rabbits are the natural reservoir of the pathogen, as they tested wild rabbits as seropositive, in which contact with domestic rabbits was virtually ruled out [57]. The partially lower seroprevalence in wild rabbits could be due to a lower population density and a consequently lower infection risk compared to domestic rabbits, as high prevalence has been found in domestic rabbits, particularly in connection with overstocking and presumably associated urine contamination [66]. An additional factor to consider that could limit the prevalence in the wild population is that wild rabbits with manifest encephalitozoonosis are most likely more susceptible to predation and, therefore, may not be among the rabbits studied [38]. This would also partially limit the possibility of diseased rabbits continuing to excrete spores, which in turn could infect other rabbits.

In the current study, *E. cuniculi* DNA was detected in the brain and kidneys of only 2 of the 24 rabbits that tested positive for antibodies using IFAT. This could occur due to very low spore concentration and/or uneven distribution in the tissue under study [45]. It is possible that the animals were still in the early phase of infection or were exposed to the pathogen but were able to fight off the infection, which may have prevented high spore numbers and their detection in the organs. Some authors report that spores are less abundant in the tissues concerned when infection progresses [19,20], which could also lead to a lower positive rate.

*E. hellem* is a potential pathogen that occurs mainly in birds and humans, but occasionally also in mammals. In Lagomorpha, both domesticated and wild, natural infection with *E. hellem* has so far only been detected in the kidney tissue of a single European brown hare, which was simultaneously infected with *E. intestinalis*. The researchers had not expected to find these pathogens, as the kidney lesions initially indicated an infection with *E. cuniculi* [27].

To the best of our knowledge, the present study presents the first successful detection of *E. hellem* in rabbits. A total of 4 of the 158 wild rabbits showed Ct values using real-time PCR. Although the Ct values are relatively high (Supplementary Table S3), the real-time PCR used is very sensitive, and no non-specific reactions between *E. hellem* and *E. cuniculi* have been documented [41]. The nested PCR used in this investigation also confirmed these results with corresponding bands in three of the four rabbits visible after agarose gel electrophoresis. For the identification of *E. hellem*, only molecular methods were used here, as there is no established antibody test for this pathogen in rabbits available. In general, knowledge of *E. hellem* in rabbits is very limited so far. The few studies published originate from Spain (including Tenerife). They were also based on molecular testing methods, and *E. hellem* could not be detected (0%, 0/383 [49], 0/50 [48], 0/438 [50]).

Further large-scale studies on this pathogen—for both domesticated and wild rabbits—could provide an important insight into the actual spread of *E. hellem* and also supply information on a possible relevance as a pathogen for rabbits.

There were some limitations of the present study that should be mentioned. The sample areas of this study were distributed as far as possible across different regions of Bavaria. However, an even distribution of samples was not achieved. Wild rabbits in Bavaria, as well as in many other states in Germany, have been eradicated or at least very severely decimated in some areas due to epidemics such as rabbit haemorrhagic disease or myxomatosis [67]. The largest proportion of the samples, therefore, originated from the Munich area, as there are still quite large wild rabbit populations in this region. Nevertheless, many counties could be included in the investigation (Figure 1).

The sample material used from the brain and kidneys is very suitable for testing for *E. cuniculi*, but *E. hellem* has so far been isolated primarily from faecal samples, even if there is also evidence from organ material [4]. It is, therefore, possible that faeces or intestinal tissue are more appropriate for the detection of this pathogen and should, therefore, be additionally included in future investigations. The inclusion of the eyes of the animals in the sample material specifically for the detection of *E. cuniculi* could possibly further increase the detection rate.

## 5. Conclusions

The results of this study show that both *E. cuniculi* and *E. hellem* are present in wild rabbits in Bavaria. This study provides the first *E. cuniculi* genotype determination in free-living wild rabbits worldwide and, in addition, the first evidence of *E. hellem* in rabbits worldwide. Wild rabbits should, therefore, be regarded as a reservoir for both pathogens and, on the basis of molecular evidence from kidney tissue and presumed urine excretion, also as a source of infection for *E. cuniculi* for animals and humans. For domestic rabbits, the possibility of infection via wild rabbits by outdoor housing or contaminated fresh feed from meadows arises. For immunocompromised persons (YOPI group) in particular, hygienic safety precautions should be considered when in contact with wild rabbits, their food products and excretions. Further studies covering larger parts of Germany would be desirable to gain deeper insights into the circulation of the pathogens in the wild rabbit population.

## Figures and Tables

**Table 1:** Age and gender of the sampled European wild rabbits and sampling year.

	Number of Rabbits
**Age**	
Juvenil	36
Adult	122
**Sex**	
Male	88
Female	70
**Sampling year**	
2021	7
2022	126
2023	25

**Table 2:** Results of indirect immunofluorescence antibody test for *E. cuniculi* with titres for IgG and IgM.

Rabbit No.	Locations (Administrative Districts)	IgG Titre	IgM Titre
2	Munich	1:80	-
3	Munich	-	1:80
9	Munich	>1:1280	1:320
13	Freising	1:640	1:80
17	Munich	1:80	-
34	Bamberg	1:160	-
37	Bamberg	1:80	-
45	Passau	1:80	1:80
46	Passau	-	1:80
52	Munich	1:1280	-
53	Munich	1:320	1:80
56	Munich	1:80	1:80
62	Munich	1:80	1:80
63	Munich	1:80	-
97	Munich	1:80	-
102	Munich	1:80	1:80
103	Munich	1:80	1:80
106	Munich	1:80	-
109	Munich	1:80	-
121	Munich	1:80	1:80
133	Ingolstadt	1:80	1:80
138	Eichstätt	1:80	1:80
145	Eichstätt	1:80	1:80
148	Eichstätt	1:80	-

## Data Availability

Data are contained within the article and Supplementary Materials. Sequence information is found in GenBank (accession numbers PQ214196 and PQ214197).

## Supplementary Materials

The following supporting information can be downloaded at https://www.mdpi.com/article/10.3390/ani14192880/s1, Table S1. Location and number of rabbits sampled from these locations; Table S2. Real-time PCR, Ct values of samples tested positive for *E. cuniculi* DNA; Table S3. Real-time PCR, Ct values of samples tested positive for *E. hellem* DNA; Figure S1. Agarose gel electrophoresis of the second round of nested PCR II showing specific PCR products of *E. cuniculi* strain (expected size about 0.3 kb); Figure S2. Agarose gel electrophoresis of the second round of nested PCR I showing specific PCR products of *E. hellem* strain (expected size about 0.3 kb).
