# Supplementary material for: Occurrence of Encephalitozoon cuniculi and Encephalitozoon hellem in European Wild Rabbits (Oryctolagus cuniculus) in Southern Germany (Bavaria)
Source: Animals (Basel). 2024 Oct 7;14(19):2880. doi: 10.3390/ani14192880 (PMC11475994; doi:10.3390/ani14192880)

Table S1. Location and number of rabbits sampled from these location

Location (Administrative district)	Number of rabbits investigated
Aichach-Friedberg	8
Ansbach	2
Aschaffenburg	20
Bamberg	17
Eichstätt	15
Erlangen	8
Freising	8
Ingolstadt	4
Landshut	2
Munich	55
Passau	2
Regensburg	1
Schwandorf	8
Schweinfurt	4
Traunstein	2
Würzburg	2

Table S2. Real-time PCR, Ct values of samples tested positive for *E. cuniculi* DNA

Rabbit no.	Ct value brain	Ct value kidneys
*9	32.91	27.67
66	-	30.92
68	-	36.15
75	38.58	-
79	38.50	-
97	-	45.98
118	40.67	39.06
134	-	34.40
155	37.66	-
157	-	38.30

* sequencing revealed genotype 1

Table S3. Real-time PCR, Ct values of samples tested positive for *E. hellem* DNA

Rabbit no.	Ct value brain	Ct value kidneys
97	-	42.93
109	44.04	-
118	47.86	48.23
150	-	43.03

Figure S1. Agarose gel electrophoresis of the second round of nested PCR II showing specific PCR products of *E. cuniculi* strain (expected size about 0.3 kb)

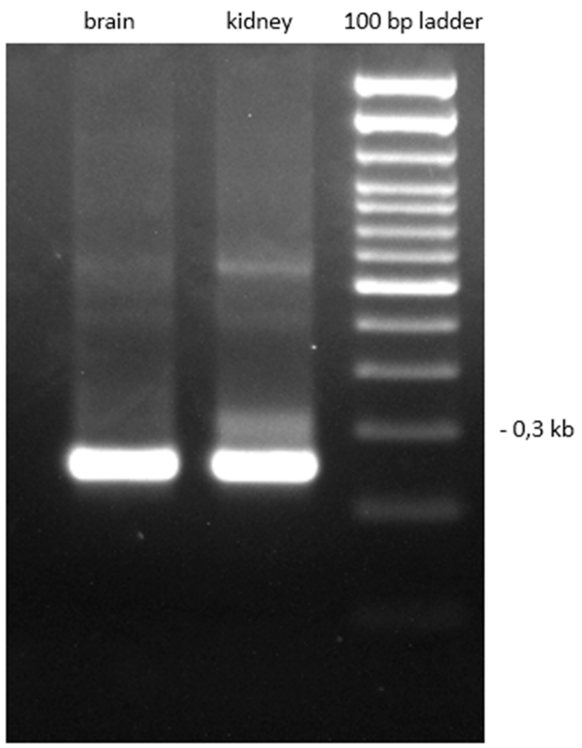

Figure S2. Agarose gel electrophoresis of the second round of nested PCR I showing specific PCR products of *E. hellem* strain (expected size about 0.3 kb)

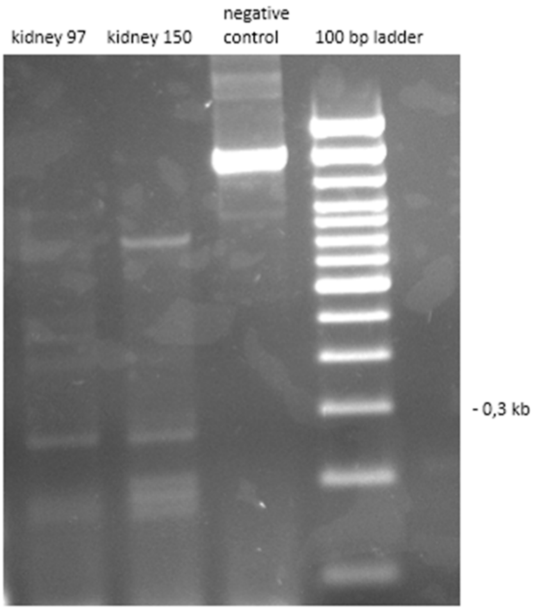

Supplement: Supplementary file 1 [file animals-14-02880-s001.zip › animals-3221388-supplementary.pdf]
